# Compartmental analysis of the pulmonary proteome reveals novel functions of the aryl hydrocarbon receptor

**DOI:** 10.1186/s12931-026-03593-7

**Published:** 2026-03-25

**Authors:** Emily T. Wilson, Nicole S. Heimbach, Roham Gorgani, Willem Rijnbout-St. James, Noof Aloufi, David H. Eidelman, Carolyn J. Baglole

**Affiliations:** 1 Department of Pharmacology and Therapeutics, McGill University, Montreal, Canada; 2 Research Institute of the McGill University Health Centre, Montreal, Canada; 3 Department of Pathology, McGill University, Montreal, Canada; 4 Department of Clinical Laboratory Sciences, Taibah University, Madinah, Saudi Arabia; 5 Department of Medicine, McGill University, Montreal, Canada; 6 RI- MUHC, Centre for Translational Biology (CTB), Block E 1001 Boul. Décarie, Montreal, QC H4A 3J1 Canada

## Abstract

As a barrier organ, the lungs are continuously exposed to environmental stimuli and rely on molecular sensors to detect and respond to these external cues. One such sensor is the aryl hydrocarbon receptor (AhR), a ligand-activated transcription factor traditionally recognized for its role in xenobiotic metabolism. However, this function is a relatively recent evolutionary adaptation of the AhR. Beyond its detoxification role, AhR has been implicated in developmental and homeostatic processes, as evidenced by defects in AhR-deficient mice. Despite this, the contribution of endogenous AhR activity to pulmonary homeostasis remains poorly defined. To address this gap, we leveraged high-resolution proteomics from lung tissue, bronchoalveolar lavage fluid (BALF), and extracellular vesicles (EVs) of AhR-expressing and AhR-knockout mice to define the role of AhR in shaping the pulmonary proteomic landscape. Our analysis reveals that each compartment exhibits a distinct proteomic signature and that AhR plays a central role dictating that composition within each compartment. Notably, AhR regulates proteins involved in epithelial barrier function, alveolar macrophage lipid handling, and the selective packaging of bioactive cargo into EVs. These findings identify AhR as a key regulator of structural and immune balance in the lung and uncover new functions of this receptor in maintaining pulmonary homeostasis. Furthermore, our work highlights the potential of EVs as sensitive indicators of AhR activity and offers new insight into therapeutic strategies targeting AhR signaling in respiratory disease.

**Supplementary Information:**

The online version contains supplementary material available at 10.1186/s12931-026-03593-7.

## Introduction

Living organisms exist in constant communication with their environment, relying on molecular sensors to detect external stimuli and coordinate adaptive responses. One of these sensors is the aryl hydrocarbon receptor (AhR), a basic helix-loop-helix (bHLH) Per-ARNT-Sim (PAS) protein that functions as a ligand-activated transcription factor. The AhR is best known for its role in xenobiotic metabolism [1]. Upon binding to exogenous small, planar, nonpolar ligands, such as the halogenated aromatic hydrocarbon dioxin, the AhR undergoes a conformational change, exposing its nuclear localization sequence and translocating into the nucleus [2]. Within the nucleus, AhR dimerizes with the aryl hydrocarbon receptor nuclear translocator (ARNT), and this heterodimer binds to xenobiotic response elements (XREs) in the promoter regions of target genes [2]. XRE binding initiates the transcription of a battery of genes, including cytochrome P450 enzymes such as CYP1A1 and CYP1B1, which hydroxylate nonpolar ligands to facilitate their excretion [2, 3].

For decades, the focus of AhR research has centered primarily on the role of the receptor in xenobiotic metabolism, a function that is a relatively recent evolutionary adaptation [4]. The AhR is an evolutionarily-conserved protein, with homologues found across metazoan species [5], indicating that it did not evolve solely to metabolize anthropogenic compounds like dioxins. Rather, this ancient protein was later coopted for xenobiotic sensing in mammals. Additional evidence to support its prominent role in mammalian physiology came from the generation of AhR-knockout mice, which exhibit pathological and developmental conditions including hepatic fibrosis, patent ductus venosus, impaired fertility, cardiac hypertrophy, and immune dysfunction [6–10]. In humans, reduced levels of AhR or impaired receptor function have been associated with a range of inflammatory and immune-mediated disorders, including autoimmune diseases [11–14]. These data support the existence of conserved endogenous roles for the AhR in tissue homeostasis [4, 15].

One of the organs with the highest AhR expression is the lungs [16, 17]. Existing evidence strongly supports that the AhR protects against environmental insults including cigarette smoke, air pollution, and infectious organisms [12, 18, 19]. As such, most research on AhR has focused on its intracellular role as a transcription factor in response to inhaled toxicants and pathogens. In this regard, compounds generated by combustion of organic matter, such as polycyclic aromatic hydrocarbons (PAHs), also activate the AhR [20, 21]; PAHs are widely present in cigarette and wildfire smoke as well as other forms of air pollution and thus are a common source of AhR ligands for most humans across the globe [22–24]. What is less well understood is the way AhR contributes to homeostatic pulmonary functions, including regulation of the pulmonary secretome. The pulmonary secretome is a collection of bioactive molecules that includes soluble proteins such as cytokines and growth factors found in bronchoalveolar lavage fluid (BALF) as well as in extracellular vesicles (EVs) [25–27]. EVs are small, membrane-bound particles released by nearly all lung cell types that contain a rich cargo of proteins, RNA transcripts, microRNAs (miRNAs), and lipids that mediate paracrine signaling [28]. These particles facilitate communication between airway and alveolar epithelial cells, immune cells, and other lung components thereby influencing vital processes such as inflammation, tissue repair, and regeneration [29]. Together, these compartments of the respiratory system (lung tissue, EVs, and BALF) not only play a central role in intercellular communication but likely reflect differences in the dynamic state of the lung microenvironment in health and disease [30]. The EV and BALF compartments can serve as less invasive compartments for sampling biological material which not only complements traditional tissue analysis but also reveals active and real-time communication networks that govern lung homeostasis or early pathological changes.

Building on these insights, we used unbiased proteomics analysis via LC–MS/MS to characterize the pulmonary secretome of mice, which includes EV cargo and the cell-free soluble protein fraction of BALF. By including analysis using AhR-deficient mice, we uncover that the AhR regulates a myriad of proteins in all three of these compartments, controlling extracellular communication through EV cargo, promoting barrier integrity, and regulating energy metabolic adaptations.

### Experimental procedures

#### Proteomics data source

This study is a secondary analysis of a previously generated, publicly reported LC–MS/MS proteomics dataset of mouse lung tissue, EVs, and BALF from *Ahr*^+/-^ and *Ahr*^-/-^ C57BL/6 mice of both sexes (3 males and 3 females per genotype) [31]. Briefly, in this study, lungs were harvested immediately after euthanasia; BALF was collected by lavaging the lungs twice with 0.5 mL PBS. Cell-free BALF was ultracentrifuged at 110,000 g for 60 min at 4 °C (TLA 120.2, Beckman Coulter Optima MAX-XP). The supernatant was retained as EV-depleted BALF, while the EV pellet was washed in PBS and subjected to a second identical ultracentrifugation before protein extraction. EVs were characterized by established methodology which included nanoparticle tracking analysis (NTA) and transmission electron microscopy TEM) in the source study. For all three compartments, proteins were run into a single stacking-gel band to remove lipids/detergents/salts, reduced with DTT, alkylated with iodoacetic acid, and digested with trypsin. Two micrograms of peptides were resuspended in 0.1% formic acid and loaded onto a Thermo Acclaim PepMap precolumn (75 µm ID × 2 cm, C18 3 µm beads) and an Acclaim PepMap EasySpray analytical column (75 µm × 15 cm, C18 2 µm beads). Peptides were separated on a Dionex Ultimate 3000 uHPLC at 250 nL/min using a 3 h gradient from 2–35% acetonitrile (0.1% formic acid) and analyzed on a Thermo Orbitrap Fusion. Raw files were converted to Mascot generic format and searched with Mascot v2.6.2 against UniProt Mus musculus (2021). Search results were imported into Scaffold Q + (Proteome Sciences; San Diego, CA).

#### Proteomics analysis

We re-analyzed these proteomics data to compare *Ahr*^+/-^ and *Ahr*^*−/−*^ mice at baseline using Scaffold Q + (Proteome Sciences; San Diego, CA). Differentially expressed proteins (DEPs) were determined using t-tests on normalized spectral values, with a significance threshold of *p*-value ≤ 0.05. ClusterProfiler (v4.10.1) in R (v4.3.2) was used to perform Gene Ontology (GO) term overrepresentation analysis [32, 33]. SEPDB was used to determine the cellular compartment of DEPs [34]. Dot plots and cnetplots were generated using enrichplot in R. DEPs involved in pathways of interest were visualized in heatmaps based on their z-scores, using GraphPad Prism (v10.2.1).

#### Lipid-laden macrophages

BAL cells were collected from *Ahr*^+/-^ and *Ahr*^*−/−*^ mice and stained with Oil Red O (Thermo Fisher Scientific; Saint-Laurent, QC). Briefly, cells were fixed in 4% paraformaldehyde for 15 min at room temperature, then washed with 70% isopropanol. The cells were stained with freshly prepared Oil Red O solution overnight, rinsed with 70% isopropanol, and washed with distilled water. Slides were counterstained with hematoxylin for 1 min, washed with distilled water, and a cover slip was applied with aqueous mounting medium. Stained cells were counted under a microscope, and the lipid laden macrophage index (LLMI) of Oil Red O-positive cells was determined independently by three individuals by counting at least 100 cells per sample and rating them from 0–4 as described previously [35].

#### Enzyme-Linked Immunosorbent Assay (ELISA)

Lipid peroxidation was assessed using a mouse Malondialdehyde (MDA) ELISA Kit (MyBioSource; San Diego, CA) according to manufacturer instructions. MDA concentrations in the samples were calculated by comparing the absorbance values to those of a standard curve generated from known MDA concentrations.

#### Actin staining

A549^WT^ and A549^AHRKO^ cells, gifted by Dr. Jason Matthews, were generated by clustered regularly interspaced short palindromic repeats (CRISPR)/Cas9 technology as previously described [36]. Cells were cultured in Dulbecco’s Modified Eagle Medium (DMEM) supplemented with 10% fetal bovine serum, 1% Glutamax, 1% antibiotic–antimycotic, and 0.1% gentamycin and incubated at 37 °C with 5% CO_2_. Cells were seeded into 8 well chamber slides (Corning, 354118; Corning, NY, USA) at a density of 4 × 10^4^ cells/ml. Cells were grown to 80% confluency before fixation with 4% paraformaldehyde (Thermo Scientific, J61899, Waltham, MA, USA) for 10 min at room temperature (RT). The cells were then permeabilized for 20 min with 0.1% Triton X-100 (Bio Basic, TB0198, Markham, Ontario, Canada). Following washing with phosphate buffered saline (PBS), cells were blocked with 3% bovine serum albumin (BSA) in PBS for 1 h at RT. The cells were then incubated for 3 h with DAPI (1:750; Thermo Scientific, Waltham, MA, USA), phalloidin AlexaFluor488 (1:400; Thermo Scientific) and deoxyribonuclease 1 AlexaFluor594 (10 µg/mL; Thermo Scientific) in PBS. Images were acquired using a Zeiss LSM780 confocal microscope (Carl Zeiss, Oberkochen, Germany). Image analysis was performed with ImageJ (Fiji) and signal threshold was normalized to experimental control. Statistical significance was determined using the Mann–Whitney test.

#### Protein concentration

Total protein concentration of BALF was determined using the Pierce™ Bicinchoninic Acid (BCA) Protein Assay Kit (Thermo Fisher Scientific; Saint-Laurent, QC), following the manufacturer’s instructions. Briefly, 25 µL of each BALF sample or bovine serum albumin (BSA) standard was added in duplicate to a 96-well microplate. Subsequently, 200 µL of the working reagent was added to each well. The plate was incubated at 37 °C for 30 min and absorbance was measured at 562 nm using a TECAN microplate reader (TECAN; Baldwin Park, CA). Protein concentrations of the BALF samples were calculated based on the standard curve generated from the BSA standards.

#### Experimental design and statistical rationale

All animal procedures were approved by the McGill University Animal Care Committee and conducted in accordance with Canadian Council on Animal Care guidelines. In vivo experiments were conducted using age-matched *Ahr*^+*/−*^ and *Ahr*^*−/−*^ mice sacrificed between 8 and 12 weeks of age. Each mouse served as an independent biological replicate. For proteomics analyses, six mice per genotype were included (*n* = 6/group), and sample types included lung tissue, BALF, and EVs. For Oil Red O quantification, BALF cells were collected from 4–5 mice per group. No animals or data points were excluded. Unpaired two-tailed t-tests were used to compare *Ahr*^+*/−*^ and *Ahr*^*−/−*^ groups for all outcomes. Proteomic pathway enrichment was based on DEPs, and z-scores were used for heatmap visualization.

## Results

### EVs represent a unique pulmonary compartment with distinct proteomic signatures

**Fig. 1:**
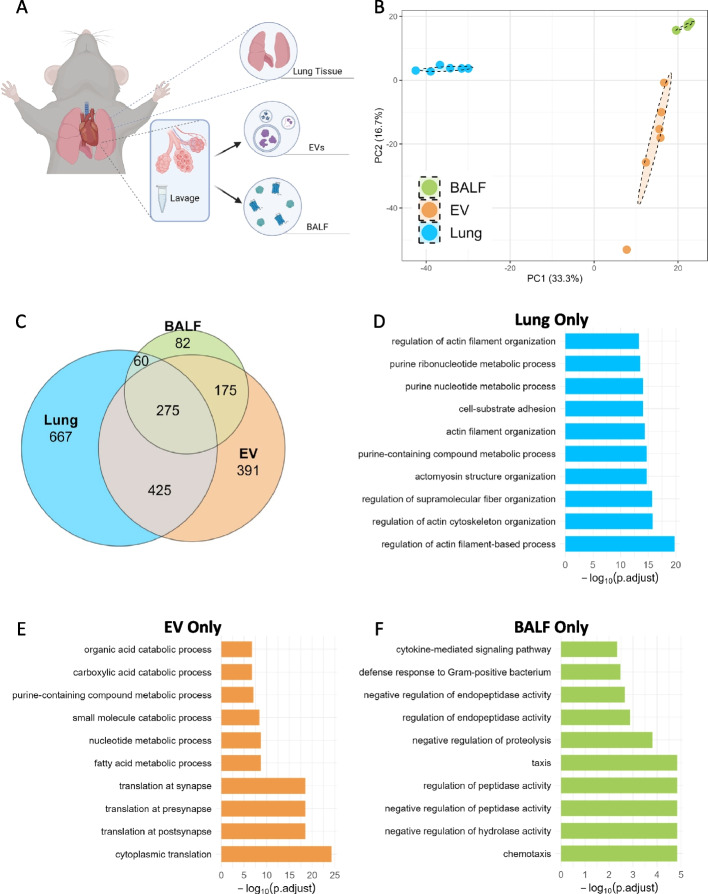
Pulmonary compartments exhibit distinct proteomic profiles. **A** Schematic representation of the lung compartments analyzed. **B** PCA of proteomic data from lung tissue, EVs, and BALF. **C** Venn diagram of identified proteins in each compartment. **D**–**F** GO term overrepresentation analysis of proteins unique to lung tissue (**D**), EVs (**E**), and BALF (**F**)

The lungs contain multiple microenvironments, each contributing to the maintenance of pulmonary function and responses to environmental exposures. Traditional assessments of lung health rely on tissue biopsies, which provide insight into structural and immune components, or BALF analysis, which captures soluble proteins and cellular debris within the airway lumen. EVs, which are membrane-bound vesicles that facilitate intercellular communication, may represent a distinct molecular compartment of the lungs; yet research into pulmonary-derived EVs remains limited. To assess whether pulmonary EVs constitute a distinct compartment that offers unique insight into lung health, we analyzed shotgun proteomics of lung tissue, EVs, and BALF collected from naïve control (*Ahr*^+/-^) mice (Fig. 1A). EVs were similar in size, concentration and morphology between genotypes as recently reported [31]. PCA revealed that each compartment has a distinct proteomic signature, with lung tissue segregating most strongly from the EVs and BALF along PC1 axis (33.3%), while EVs and BALF were more closely related, differentiating along the PC2 axis (16.7%; Fig. 1B). These findings suggest that EVs are compositionally distinct from both lung tissue and BALF, potentially reflecting unique proteins not captured by traditional lung sampling approaches.

To further understand the compartment-specific proteomes, we compared proteins detected in each compartment (Fig. 1C, Supp. Table 1). Lung tissue contained 667 unique proteins, EVs contained 391 unique proteins, and BALF contained 82 unique proteins. To determine the functional significance of these compartment-specific proteins, we performed GO overrepresentation analysis (Supp. Tables 2–4). Proteins found only in lung tissue were involved in extracellular matrix (ECM) organization and nucleotide metabolism, both processes essential for maintaining pulmonary architecture and mitochondrial function (Fig. 1D). In contrast, EV-exclusive proteins were involved in translational and metabolic processes, including cytoplasmic translation and lipid metabolism, suggesting that EVs actively contribute to protein synthesis and metabolic regulation within the lung environment (Fig. 1E). Lastly, BALF-exclusive proteins were associated with immune signaling, growth factors, and extracellular remodeling, reinforcing the role of BALF components in host defense and airway maintenance (Fig. 1F). Together, these findings demonstrate that EVs represent a biologically distinct compartment within the lung, complementing insights gained from lung tissue and BALF.

### AhR-dependent control over proteins in lung tissue, EVs, and BALF

**Fig. 2:**
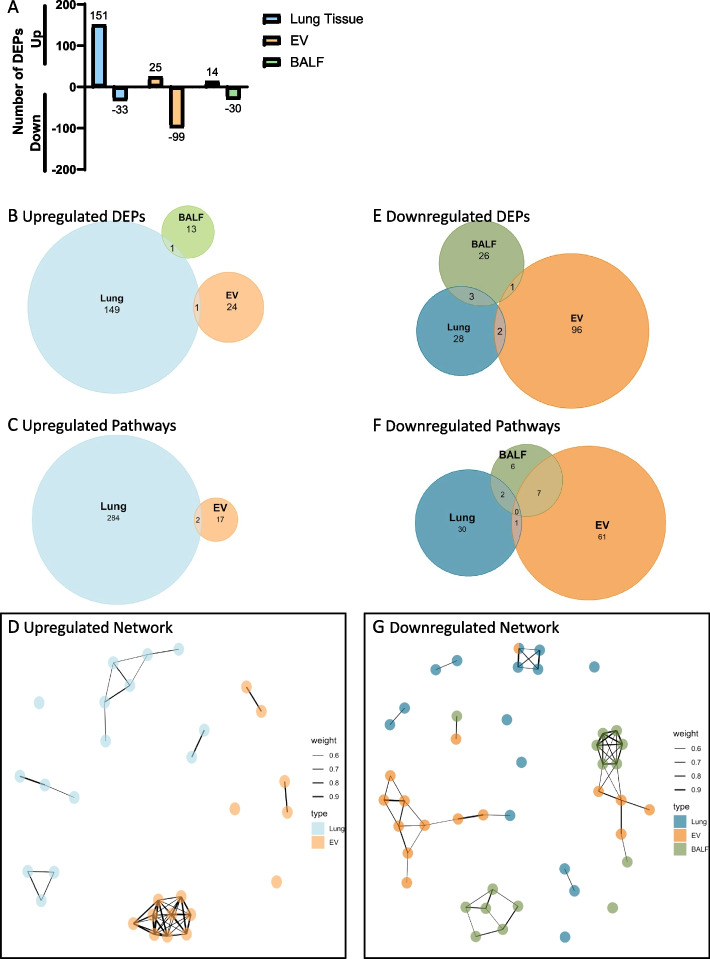
Compartment-specific protein regulation by AhR in the pulmonary system. **A** DEPs regulated by AhR across lung tissue, EVs, and BALF, with upregulated proteins (positive values) and downregulated proteins (negative values). **B** Venn diagram depicting the distribution of upregulated DEPs across compartments. **C** Venn diagram of upregulated pathways, showing the overlap between lung tissue and EVs. **D** Network representation of top 15 upregulated pathways for each compartment, with nodes representing pathways and edges indicating shared functional relationships. **E** Venn diagram illustrating downregulated DEPs and their distribution across compartments. **F** Venn diagram of downregulated pathways between lung tissue, EVs, and BALF. **G** Network analysis of top downregulated pathways from lung tissue, EVs, and BALF. Additional network analysis can be found in the online supplement

AhR is ubiquitously-expressed in the lungs and is essential for maintaining respiratory health [37]. Most pulmonary cells express AhR, including epithelial and endothelial cells, fibroblasts and macrophages [12, 19, 38–40] (Supp. Figure 1). However, its endogenous functions within the pulmonary system remain incompletely characterized. To better clarify the role of the AhR under steady-state conditions, DEPs were identified in lung tissue, EVs, and BALF of *Ahr*^+/-^ mice vs *Ahr*^*−/−*^ mice (Supp. Table. 5–7). Therefore, data are presented as being up-or-down-regulated in relation to *Ahr*^+/-^ mice unless otherwise specified. These data show that the AhR regulates protein expression across lung compartments, selectively enhancing protein expression in lung tissue while suppressing proteins in EVs and BALF (Fig. 2A). For the upregulated proteins, AhR enhanced protein expression within the lung tissue (Fig. 2B, Supp. Table 8). Interestingly, lung tissue and EVs only shared one AhR-dependent protein, H4c1, a nucleosome component that is elevated in EVs during cellular stress [41]. To understand the potential impact of these upregulated DEPs, we performed GO term overrepresentation analysis. Of the compartments analyzed, lung tissue had the most extensive AhR-dependent pathways, with 286 pathways that were significantly upregulated. EVs showed a limited response with 19 upregulated pathways, and BALF exhibited no significant enrichment (Fig. 2C, Supp. Table 9). Lung tissue and EVs shared two pathways: response to metal ion and response to salt (Supp. Table 9). Network analysis of the top 15 upregulated pathways from each compartment further revealed compartment-specific clustering, suggesting that AhR promotes distinct pathways within each compartment rather than promoting proteins that have overlapping roles (Fig. 2D, Supp. Figure 2).

Analysis of proteins suppressed by AhR shows that most are localized within EVs (Fig. 2E, Supp. Table 10). The largest overlap of downregulated proteins occurred between lung tissue and BALF, which shared Hspa8, Idh2, and Pdia6, proteins that are involved in protein folding and metabolic stress responses [42–44]. Overrepresentation analysis indicated that in the absence of AhR, EVs have the largest number of enriched pathways (Fig. 2F, Supp. Table 11). The greatest overlap of pathways was between EVs and BALF and was related to fatty acid and nucleotide metabolism. Network analysis further revealed that EVs and BALF cluster together with pathways related to metabolic processes, while EVs and lung tissue share pathways related to vesicle budding and cellular detoxification (Fig. 2G, Supp. Figure 3). Overall, these data suggest that AhR modulates protein expression across lung tissue, EVs, and BALF, enhancing expression in lung tissue while inhibiting protein packaging into EVs. Each compartment exhibits unique functions with minimal overlaps, highlighting the compartment-specific roles of AhR in pulmonary homeostasis.

### AhR supports the expression of proteins involved in cell adhesion and maintenance of tissue architecture while suppressing detoxification pathways

**Fig. 3:**
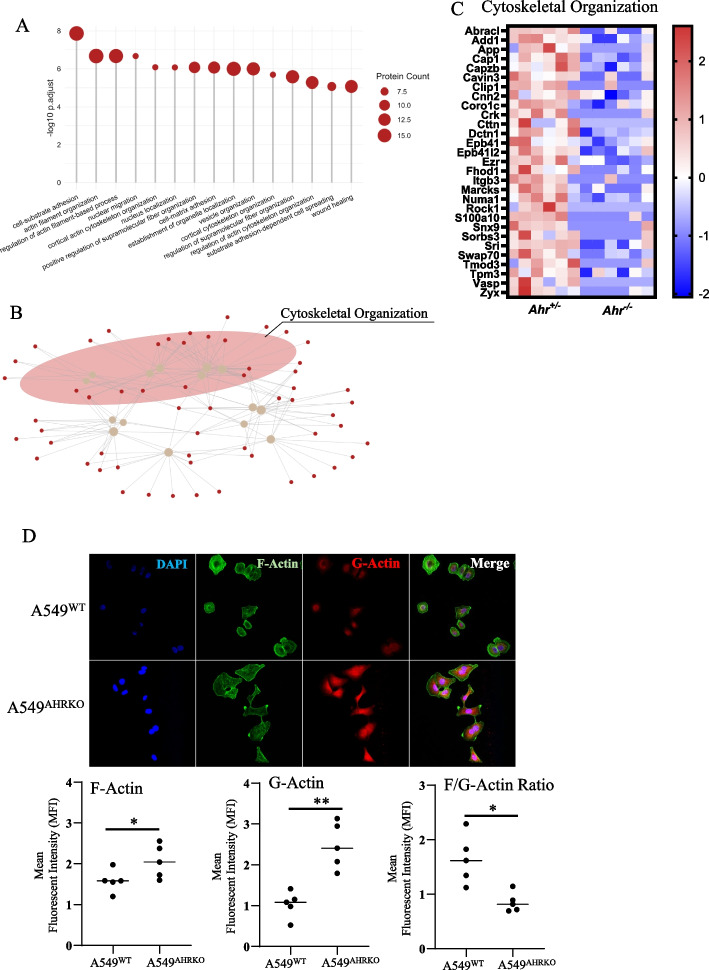
Upregulated DEP pathways in lung tissue from *Ahr*^+/-^ mice highlights cytoskeletal organization. **A**-**B** Overrepresentation analysis of upregulated regulated GO pathways in *Ahr*^+/-^ vs *Ahr*^*−/−*^ mice visualized as a dot plot (**A**) and network plot showing the overlap of proteins between pathways (**B**). Heatmap of proteins contributing to the top five upregulated pathways, colour represents z-score, shows changes in proteins involved in cytoskeletal organization (**C**). Staining of actin structures in A549^WT^ and A549^AHRKO^ cells. Note the significant changes in F- and G actin. *N* = 5 independent replicates; *p < 0.05; **p < 0.01 (**D**)

In the lung tissue, presence of AhR correlated with an increased abundance of proteins involved in tissue architecture, particularly pathways related to cell adhesion, actin dynamics, and cytoskeletal organization (Fig. 3A). The largest pathway cluster in network analysis was related to cytoskeletal organization (Fig. 3B, Supp. Figure 4). Expression of individual DEPs associated with actin cytoskeletal structure was significantly higher in *Ahr*^+/-^ lung tissue, including Rock1, Fhod1, and Cttn (Fig. 3C). These proteins regulate stress fiber formation, actin filament nucleation, and branched actin network assembly, respectively [45–47]. Their upregulation in *Ahr*^+/-^ murine lung tissue suggests that AhR plays a central role in cytoskeletal maintenance. Therefore, we used the human alveolar type II epithelial model, A549 cells, in which AhR was knocked out, to evaluate epithelial cell integrity and morphology. Here, A549^AHRKO^ cells exhibited actin morphological changes including actin reorganization and dysregulation in the F/G actin ratio (Fig. 3D) This phenotype is consistent with the downregulation of Rock1, Fhod1, Cttn, Vasp, and Capzb in our proteomics data and underscores the importance of AhR in maintaining actin cytoskeletal integrity.

**Fig. 4:**
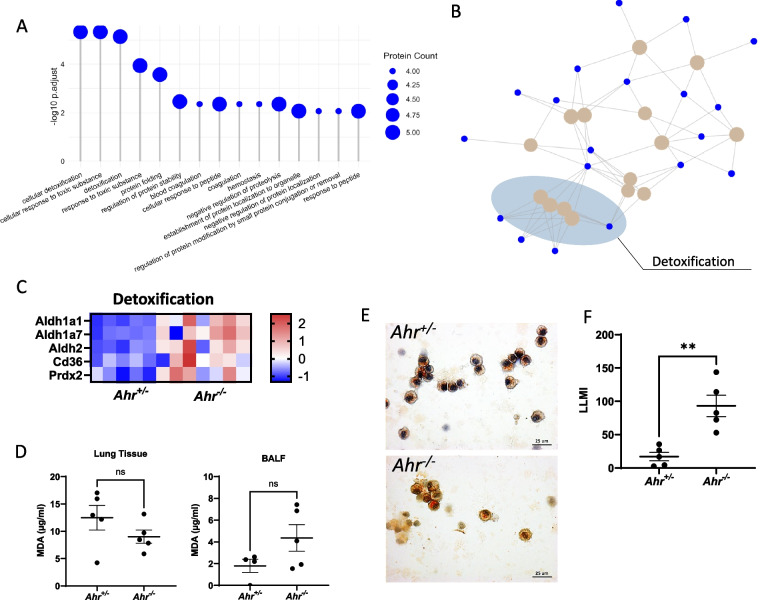
Downregulated DEP pathway analysis in lung tissue of *Ahr*^+/-^ and *Ahr*^*−/−*^ mice. **A**-**B** Overrepresentation analysis of downregulated regulated GO pathways in *Ahr*^+/-^ vs *Ahr*^*−/−*^ mice, visualized as a dot plot (**A**) or network plot showing the overlap of proteins between pathways (**B**). Heatmap of proteins contributing to the detoxification pathway cluster, colour represents z-score (**C**). Concentration of MDA in lung tissue and BALF of *Ahr*^+/-^ and *Ahr*^*−/−*^ mice (**D**). **E**–**F** Oil red O staining of BAL cells (**E**) and quantification of lipid-laden macrophages (**F**)

Proteins downregulated by AhR in the lung tissue were involved in detoxification pathways (Fig. 4A). The most downregulated pathways formed a tight cluster and shared the same five proteins (Fig. 4B, Supp. Figure 5), including Aldh1a1, Aldh1a7, and Aldh2 (Fig. 4C). Aldh2 directly oxidizes MDA, a byproduct of lipid peroxidation and a marker of oxidative stress [48]. To assess if there was a corresponding functional impact with the decrease of proteins important in detoxification, we measured MDA levels in lung tissue and BALF of *Ahr*^+/-^ and *Ahr*^*−/−*^ mice. Although BALF from *Ahr*^*−/−*^ mice showed a slight increase in MDA, the overall MDA levels in both lung tissue and BALF were not significantly altered (Fig. 4D). Another protein that was decreased in lung tissue from *Ahr*^+/-^ mice was Cd36, a scavenger receptor that mediates lipid uptake by alveolar macrophages [49]. We therefore examined lipid accumulation in alveolar macrophages isolated from the BAL of the mice and found that *Ahr*^*−/−*^ mice, which exhibited higher Cd36 expression, also showed an increased proportion of lipid-laden alveolar macrophages (LLAMs; Fig. 4E-F). This observation highlights a novel role of AhR in contributing to lipid metabolism in alveolar macrophages. Collectively, these results underscore that the endogenous functions of AhR in the lung are to balance structural integrity, participate in redox regulation, and promote lipid homeostasis.

### AhR modulates EV cargo to promote barrier integrity and suppress detoxification

**Fig. 5:**
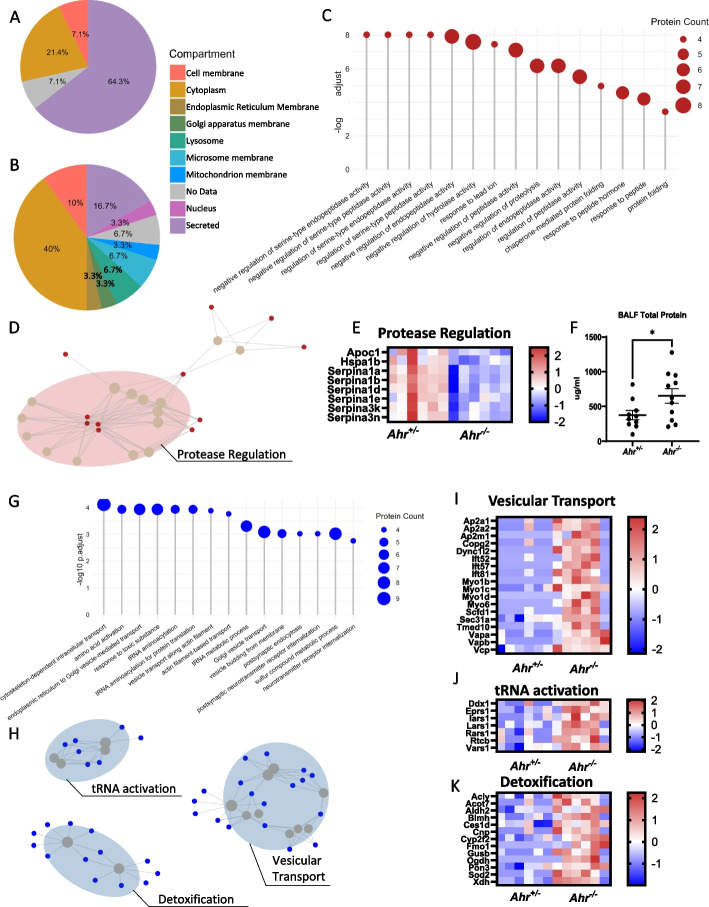
Pathway analysis of DEPs in EVs of *Ahr*^+/-^ and *Ahr*^*−/−*^ mice. **A**-**B** SEPDB analysis of upregulated (**A**) and downregulated (**B**) DEPs in EVs of *Ahr*^+/-^ vs *Ahr*^*−/−*^ mice. **C**-**D** Overrepresentation analysis of positively regulated GO pathways visualized as a dot plot (**C**) and network plot showing the overlap of proteins between pathways (**D**). Heatmap of proteins contributing protease regulation cluster, colour represents z-score (**E**). Total protein in BALF of *Ahr*^+/-^ and *Ahr*^*−/−*^ mice (**F**). **G**-**H** Overrepresentation analysis of negatively regulated GO pathways in *Ahr*^+/-^ vs *Ahr*^*−/−*^ mice, visualized as a dot plot (**G**) and network plot showing the overlap of proteins between pathways (**H**). **I**-**K** Heatmaps of proteins contributing to each pathway cluster including transport (**I**), tRNA aminoacylation (**J**), and detoxification (**K**). Colour represents z-score

Given the potential importance of the secretome in lung health and disease, we next investigated how AhR influences EV cargo in the pulmonary system. We characterized proteins that were preserved or suppressed by AhR by cross-referencing our data with the SEPDB (Supp. Figure 6). Interestingly, in the absence of AhR, there was an increased proportion of EV proteins that are typically localized to the cytoplasm or intracellular organelles such as the endoplasmic reticulum, nucleus, and mitochondrial matrix (Fig. 5A-B). The proteins that AhR promoted in EVs were involved in serine-type peptidase and endopeptidase activity (Fig. 5C). These pathways involved eight proteins (Fig. 5D, Supp. Figure 7 ), including four alpha-1-antitrypin (A1AT) variants (Serpina1a, Serpina1b, Serpina1d, and Serpina1e). A1AT, the enzyme that prevents proteolytic destruction of lung tissue by neutrophil elastase, was highly expressed in the EVs of *Ahr*^+/-^ mice compared to *Ahr*^*−/−*^ mice (Fig. 5 E). It is noteworthy that A1AT deficiency is the only known genetic cause of chronic obstructive pulmonary disease (COPD) [50], and to our knowledge, this is the first report showing that AhR regulates these A1AT variants. Decreased A1AT activity can weaken alveolar-capillary barrier integrity over time [51], raising the possibility that decreased expression of this enzyme in *Ahr*^*−/−*^ EVs could be associated with impaired barrier function. To assess this, we measured BALF protein concentration, a surrogate marker of barrier integrity, which revealed that *Ahr*^*−/−*^ mice exhibited higher total protein levels than *Ahr*^+/-^ mice (Fig. 5F). This indicates that there is greater lung barrier disruption in *Ahr*^*−/−*^ mice and thus supports the role of AhR in maintaining lung structural integrity.

Finally, downregulated proteins in the EVs of *Ahr*^+/-^ mice were primarily associated with vesicular transport, translation, and response to toxic substances (Fig. 5G-H, Supp. Figure 8). Notably, proteins involved in vesicle-mediated intracellular transport, such as COPII complex components (Sec31a, Scfd1) and coatomer subunits (Copg2), were suppressed by AhR, suggesting that AhR may inhibit EV release (Fig. 5I). The AhR also suppressed aminoacyl-tRNA synthetases (Eprs1, Iars1, Lars1, Rars1, and Vars1), enzymes that link tRNAs to their respective amino acids during translation (Fig. 5J). Lastly, similar to lung tissue, AhR suppressed various detoxification proteins in EVs (Fig. 5K), including Cyp2f2, a cytochrome P450 isoform that metabolizes toxic chemicals [52]. These novel findings demonstrate that AhR regulates EV cargo composition even in the absence of exogenous stimuli, promoting protease inhibitor expression while suppressing vesicular transport and detoxification pathways. This regulation is essential for maintaining pulmonary barrier integrity and shaping the response to environmental toxicants.

### AhR regulates metabolic pathways in BALF

**Fig. 6:**
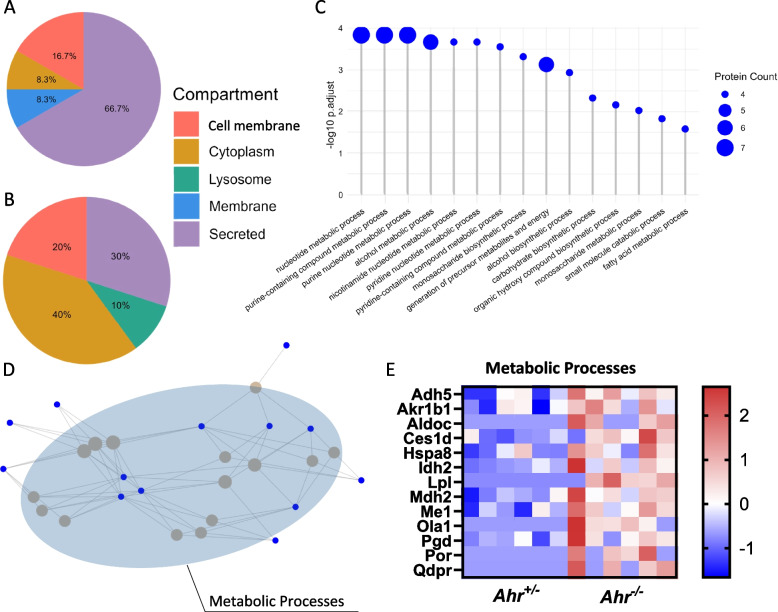
Pathway analysis of DEPs in BALF of *Ahr*^+/-^ and *Ahr*^*−/−*^ mice. **A**-**B** SEPDB analysis of upregulated (**A**) and downregulated (**B**) DEPs in BALF of *Ahr*^+/-^ vs *Ahr*^*−/−*^ mice. **C**-**D** Overrepresentation analysis of negatively regulated GO pathways in *Ahr*^+/-^ vs *Ahr*^*−/−*^ mice visualized as dot plot (**C**) and network plot showing the overlap of proteins between pathways (**D**). Heatmap of proteins contributing metabolic processes (**E**). Colour represents z-score

The last pulmonary compartment we investigated was BALF, which contains the soluble proteins released by cells. These proteins primarily originate from epithelial cells, alveolar macrophages, or vascular leakage from the bloodstream, reflecting both local cellular activity and alveolar-capillary integrity [53]. Analysis of BALF protein origins using SEPDB (Supp. Figure 9) revealed that in the presence of AhR, most proteins were classified as secreted (Fig. 6A). However, in the absence of AhR, there was a substantial increase in the proportion of BALF proteins typically localized to the cytoplasm, cell membrane, and lysosomes (Fig. 6B). This suggests that AhR deficiency may lead to increased cell necrosis or heightened susceptibility to cell death. Overall, AhR had a predominately suppressive role in BALF as there were no upregulated pathways in this compartment. The top downregulated pathways in BALF of *Ahr*^+/-^ mice encompassed various metabolic processes, suggesting that AhR modulates energy balance and nucleotide turnover within the lung (Fig. 6C). These metabolic pathways formed a tight functional network (Fig. 6D, Supp. Figure 10), and included proteins such as Adh5 and Por (Fig. 6E) which play a role in alcohol metabolism, a process that is known to be upregulated in the BALF of COPD patients [54]. These findings suggest that AhR regulates BALF protein composition and metabolic homeostasis. Collectively, our results position AhR as a regulator of pulmonary homeostasis, coordinating barrier integrity, alveolar macrophage lipid metabolism, and metabolic networks across lung tissue, EVs, and BALF.

## Discussion

Understanding the steady-state processes involved in pulmonary homeostasis is essential for deciphering how the lungs respond to external stimuli and the factors that can contribute toward either health or disease. While AhR is traditionally recognized as an environmental sensor, research has highlighted its endogenous roles in physiology [55]. The lung is a heterogeneous organ, consisting of multiple lobes, over 40 distinct cell types, and various compartments that work together to regulate pulmonary functions [56, 57]. One of these compartments is EVs, which are an important component of intercellular communication, providing insight into cellular state and disease progression [57, 58]. We establish that EVs possess a unique proteomic signature compared to lung tissue and BALF. Moreover, we demonstrate unique functions of the lung tissue and pulmonary secretome (EVs and BALF) that are controlled by the AhR. Our findings are the first to show that the AhR plays a pivotal role across pulmonary compartments by preserving lung barrier integrity, supporting alveolar macrophage lipid homeostasis, and regulating cargo packaging in EVs.

A central finding of this work is that AhR plays a fundamental role in maintaining lung barrier integrity, a critical function of this external-facing organ that protects the body from foreign insults such as pollutants, pathogens, and other inhaled toxicants [59]. An important process supporting barrier integrity is cytoskeletal organization, which contributes to epithelial cell shape, adhesion, and the mechanical stability required to preserve barrier function [27, 60]. AhR enhances cytoskeletal organization in lung tissue by upregulating actin-associated proteins such as Rock1, Fhod1, and Cttn, which are key regulators of stress fiber formation and cytoskeletal remodeling [61–63]. Defective cytoskeletal organization affects epithelial integrity and repair, which can lead to barrier dysfunction, increased permeability, and chronic inflammation [64]. Our data that there is an imbalance in the actin cytoskeleton in A549^AHRKO^ cells is consistent with previous work in other cell types [65–68]. This work is the first to demonstrate that AhR drives the expression of Vasp, which catalyzes G-actin addition during F-actin fibrillization, and Capzb, which maintains filament capping [69, 70]. Loss of AhR disrupts F-actin assembly and increases the G-actin pool, consistent with impaired G-actin incorporation into filaments. In addition to this intracellular role, AhR also exerts control at the extracellular level by regulating the packaging of protease inhibitors into EVs, most notably A1AT. A1AT is a critical antiprotease that neutralizes neutrophil elastase during inflammatory responses [71]. As A1AT is primarily synthesized in the liver and delivered to the lung via circulation [71], its enrichment in EVs suggests a potential AhR-dependent mechanism to enhance local delivery or stability. This is particularly relevant as EVs can protect cargo from enzymatic degradation [72], potentially enabling more efficient antiprotease defense within the lung microenvironment. Interestingly, A1AT deficiency is a well-established genetic cause of early-onset emphysema, characterized by unchecked protease activity and lung structural degradation leading to airflow obstruction [73]. We have previously published that *Ahr*^*−/−*^ mice exhibit persistent pulmonary neutrophilic inflammation, decreased lung function, and concomitant airspace enlargement (the murine surrogate of emphysema) in response to chronic cigarette smoke [12, 40, 74], the main cause of COPD. When considered together, this suggests that the AhR exerts multifaceted control over pulmonary homeostasis. Consistent with this notion, there was also an increase in BALF total protein levels in *Ahr*^*−/−*^ mice, which indicates greater alveolar-capillary leakage and impaired barrier integrity. Historically, the role of AhR on barrier integrity has been largely explored in the context of the intestinal tract, where it functions to maintain epithelial homeostasis and regulate immune responses [75]. In this context, microbial and dietary ligands activate AhR to promote IL-22 production and tight junction protein expression, thereby reinforcing barrier function and protecting against inflammation [75]. Recent research has also shown that AhR activation is essential for preserving epithelial integrity in primary mouse alveolar epithelial cells [76]. Overall, these data identify AhR as a central regulator of pulmonary barrier function, with potential implications in diseases for which barrier dysfunction is a hallmark feature, such as COPD, acute respiratory distress syndrome, and asthma [77–79].

While maintenance of the structural barrier is an important innate defense mechanism, the lungs are also equipped with resident innate immune cells that serve as an important line of defense against inhaled pathogens and particulates [80]. Among these, alveolar macrophages play a critical role in clearing foreign material, resolving inflammation, and maintaining surfactant homeostasis [81]. Pulmonary surfactant is a lipid-protein complex critical for alveolar stability and must be tightly regulated to prevent respiratory collapse [82]. A key pathway in surfactant turnover involves the scavenger receptor Cd36, which facilitates lipid uptake by macrophages [49]. Disruption of lipid uptake in alveolar macrophages can lead to LLAMs, a dysfunctional macrophage phenotype marked by impaired lipid handling and chronic inflammation [83]. The accumulation of LLAMs is frequently observed in chronic lung diseases, including COPD, where their abundance correlates with reduced lung function and defective clearance of pathogens [83]. Additionally, LLAMs exhibit deficient phagocytic capacity, impairing their ability to clear pathogens and contributing to persistent respiratory infections [84, 85]. It is therefore notable that AhR inhibits the formation of LLAMs, possibly through the AhR-dependent suppression of Cd36. These findings build on previous work demonstrating that AhR is required for proper macrophage phagocytosis [86], and provide a mechanistic explanation for why *Ahr*^*−/−*^ mice are more susceptible to respiratory infections [87]. These results suggest that individuals with impaired AhR function, whether from chronic exposures like cigarette smoke or genetic polymorphisms, may be more vulnerable to respiratory infections and LLAM-associated conditions.

Beyond direct immune defense, the lung also relies on complex intercellular communication networks to maintain homeostasis. Cellular communication in the lungs is mediated through direct cell–cell contact as well as a range of secreted factors, including cytokines, chemokines, metabolites, and other secretory proteins [88]. These factors are captured by BALF collection in patients and can give insight into immune activation, tissue repair, and disease progressions [89, 90]. For example, elevated levels of interleukins can be an indicator of infection [91], and in diseases like lung cancer, the detection of soluble mediators such as TGF-β and VEGF-A in BALF can aid in diagnosis and provide insight into disease status [92]. Another signaling modality is the EVs, which transport proteins, lipids, and nucleic acids between cells [93]. Our findings suggest that pulmonary EVs represent a distinct molecular compartment enriched in components of the translational machinery, including ribosomal proteins and aminoacyl-tRNA synthetases. The presence of these proteins suggests that EVs may actively support or initiate translation in recipient cells, thereby amplifying the biological impact of delivered RNA and protein cargo. Recent research supports this concept, demonstrating that airway-derived EVs are enriched in translational machinery even under basal conditions, suggesting a constitutive role in supporting protein synthesis and cellular responsiveness [94]. This function may be particularly relevant during injury or stress, where recipient cells must rapidly adjust their proteome without relying on de novo transcription. Dysregulated EV signaling has been implicated in lung pathologies including COPD, pulmonary fibrosis, and acute lung injury [58].

To the best of our knowledge, we are the first to directly examine the role of AhR in EV cargo composition under baseline conditions. While AhR has been previously implicated in EV biogenesis and secretion, this has largely been demonstrated in the context of exogenous ligand exposure. Polycyclic aromatic hydrocarbons (PAHs) are a class of environmental contaminants and well-characterized AhR ligands that are produced during the combustion of organic material and as such, are present in sources such as cigarette smoke and wildfire-related air pollution [95]. PAHs induce EV release in hepatocytes, with secretion levels corresponding to AhR-ligand affinity, as higher affinity ligands induced a larger EV response [96]. Our data reveal that AhR also regulates the composition of pulmonary EVs even in the absence of exogenous stimuli. This points to a previously unrecognized role for the AhR in controlling cargo packaging of EVs. In the absence of AhR, EVs were enriched with proteins originating from the cytoplasm as well as various organelles including the Golgi, endoplasmic reticulum, and mitochondria. One explanation for this is that *Ahr*^*−/−*^ cells experience heightened stress, such as ER stress, oxidative stress, or mitochondrial dysfunction, leading to an increased export of misfolded, damaged, or excess proteins into EVs as a protective mechanism. Alternatively, AhR could regulate protein sorting and retention in organelles, and its absence therefore disrupts vesicle biogenesis, resulting in indiscriminate cargo packaging. Supporting this notion, AhR downregulated multiple vesicle transport proteins in EVs, including Ap2, Copg2, Scfd1, Sec31a, Vapa, and Vcp. Without AhR, these trafficking components are upregulated, potentially altering sorting and export pathways, leading to the enrichment of cytosolic and organelle-derived proteins in EVs. These results position AhR as a central regulator of EV cargo composition, implicating the receptor in intercellular stress signaling, and potential therapeutic targeting for disorders characterized by EV-mediated dysfunction such as COPD [97].

While this study provides important insights into the compartment-specific endogenous roles of AhR in pulmonary homeostasis, several limitations should be considered. One limitation is that although AhR is known to respond to a range of endogenous ligands, such as tryptophan metabolites, this study did not assess the role of endogenous ligand signaling on AhR-mediated outcomes. Another consideration is that while we identified compartment-specific proteomic signatures controlled by the AhR, we are unable to assign these proteomic changes to specific cell types. The lung is composed of a complex mixture of structural and immune cells [96], and cell-type-specific contributions to proteomic alterations, especially with regards to EV production and cargo, remain unclear. The use of a CD9truc-EGFP reporter mouse crossed with cell-type-specific Cre drivers on an AhR-deficient background would allow for the identification and characterization of EVs secreted by distinct lung cell populations [98]. Such approaches would enable a more precise understanding of the cell type-specific mechanisms through which AhR regulates EV packaging, advancing our knowledge of its role in pulmonary intercellular communication and homeostasis.

In summary, our data indicate that the AhR is a central regulator of lung homeostasis through multiple interrelated mechanisms. At the cellular level, AhR enhances cytoskeletal organization to preserve epithelial barrier integrity, while at the extracellular level, it modulates the composition of EV and BALF proteins. These findings expand the scope of AhR biology to include constitutive regulation of vesicle trafficking and suggests that loss of AhR function may predispose individuals to diseases characterized by barrier disruption, immune dysfunction, and aberrant intercellular signaling. Our work positions AhR as a potential target for modulating immune and structural balance in the lung, and highlights EV composition as a window into the functional consequences of AhR deficiency.

## Supplementary Information

Supplementary Material 1.

Supplementary Material 2.

## Data Availability

All data are available within this article, in the online supplement, deposited at https://figshare.com/s/42d3632a8f837413ff80, and from the corresponding author upon reasonable request.
