# Supplementary material for: Compartmental analysis of the pulmonary proteome reveals novel functions of the aryl hydrocarbon receptor
Source: Respir Res. 2026 Mar 25;27:266. doi: 10.1186/s12931-026-03593-7 (PMC13321735; doi:10.1186/s12931-026-03593-7)
Supplement: Supplementary file 2 — Supplementary Material 2. [file 12931_2026_3593_MOESM2_ESM.pdf]

Online Supplement

Compartmental Analysis of the Pulmonary Proteome Reveals Novel Functions of the Aryl Hydrocarbon Receptor

Emily T. Wilson, Nicole Heimbach, Roham Gorgani, Willem Rijnbout-St. James, Noof Aloufi, David H. Eidelman, and Carolyn J. Baglole

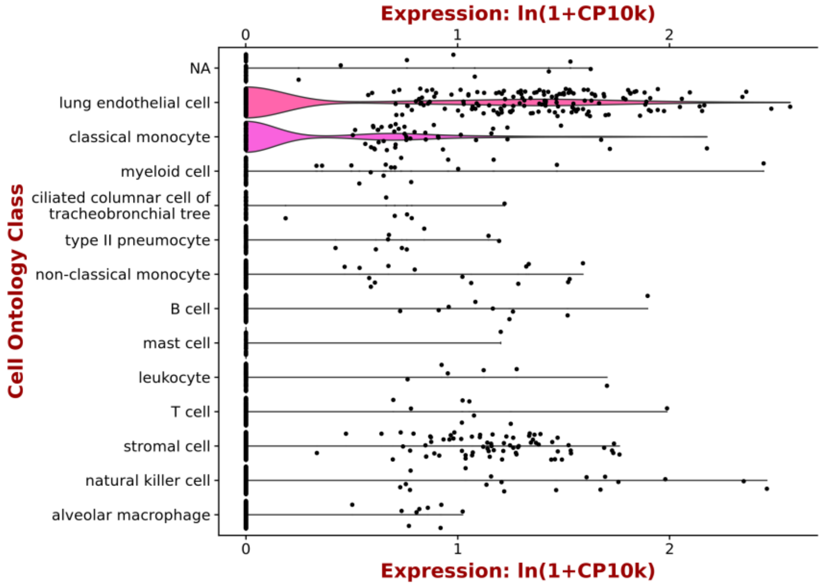

Tissue	Mean	Median	Median > 0	# cells	% 0	% > 0
lung endothelial cell	0.45	0	1.40	462	67.53	32.47
classical monocyte	0.23	0	0.75	161	73.29	26.71
NA	0.18	0	1.03	45	82.22	17.78
myeloid cell	0.16	0	0.69	87	81.61	18.39
ciliated columnar cell of tracheobronchial tree	0.09	0	0.73	49	87.76	12.24
type II pneumocyte	0.08	0	0.74	89	89.89	10.11
non-classical monocyte	0.07	0	1.02	220	93.18	6.82
B cell	0.05	0	1.17	204	95.59	4.41
mast cell	0.05	0	1.20	24	95.83	4.17
leukocyte	0.04	0	1.04	152	96.05	3.95
T cell	0.04	0	1.04	247	96.76	3.24
stromal cell	0.03	0	1.16	2540	97.17	2.83
natural killer cell	0.03	0	1.22	824	97.94	2.06
alveolar macrophage	0.02	0	0.82	345	97.39	2.61

Supp Figure 1. Expression of *Ahr* in mouse lung cell populations (from https://tabula-muris.ds.czbiohub.org/); original publication can be found here: https://doi.org/10.1038/s41586-018-0590-4.

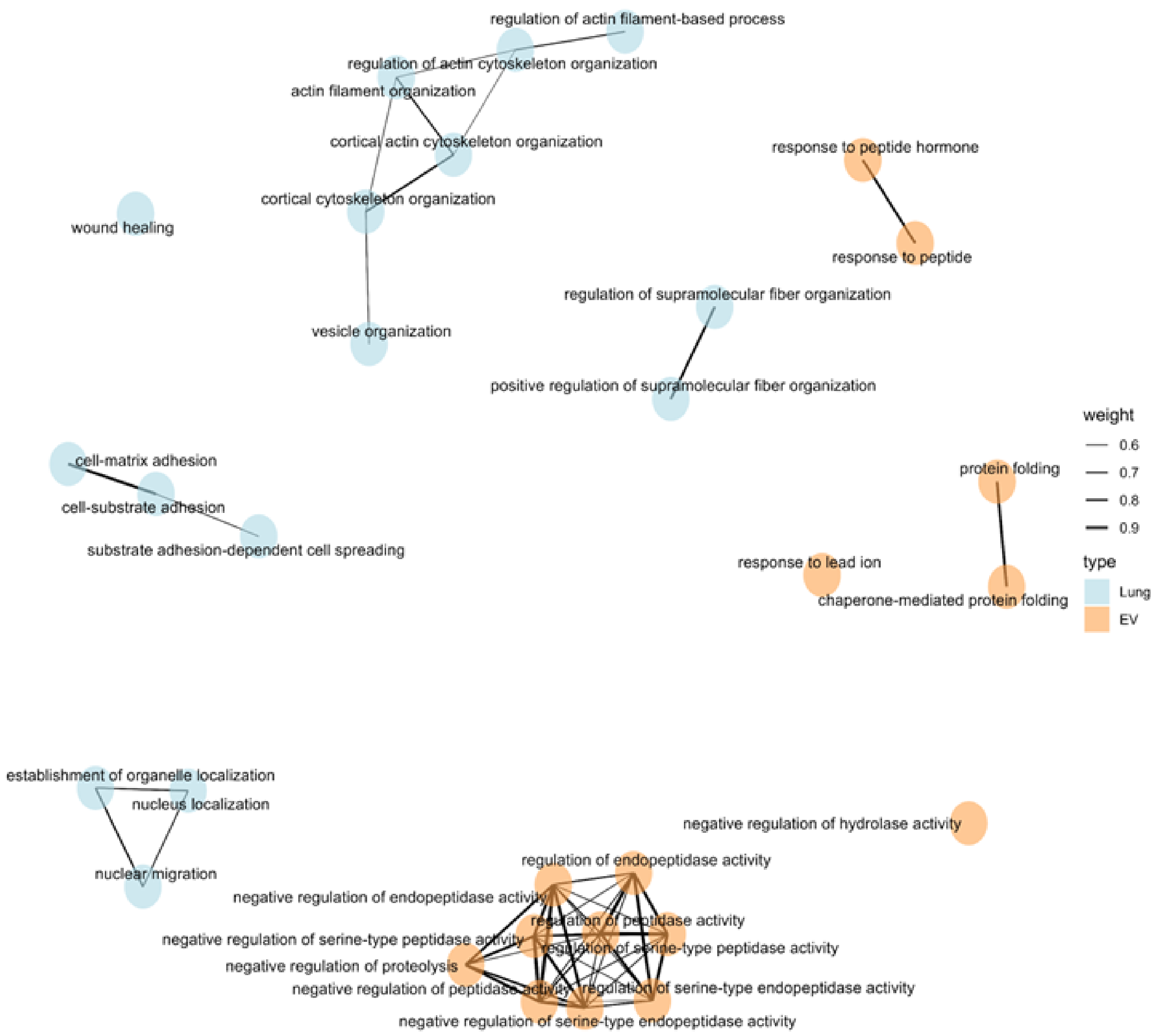

Supp. Fig 2. Labelled network analysis of top 15 upregulated pathways for each compartment, with nodes representing pathways and edges indicating shared functional relationships.

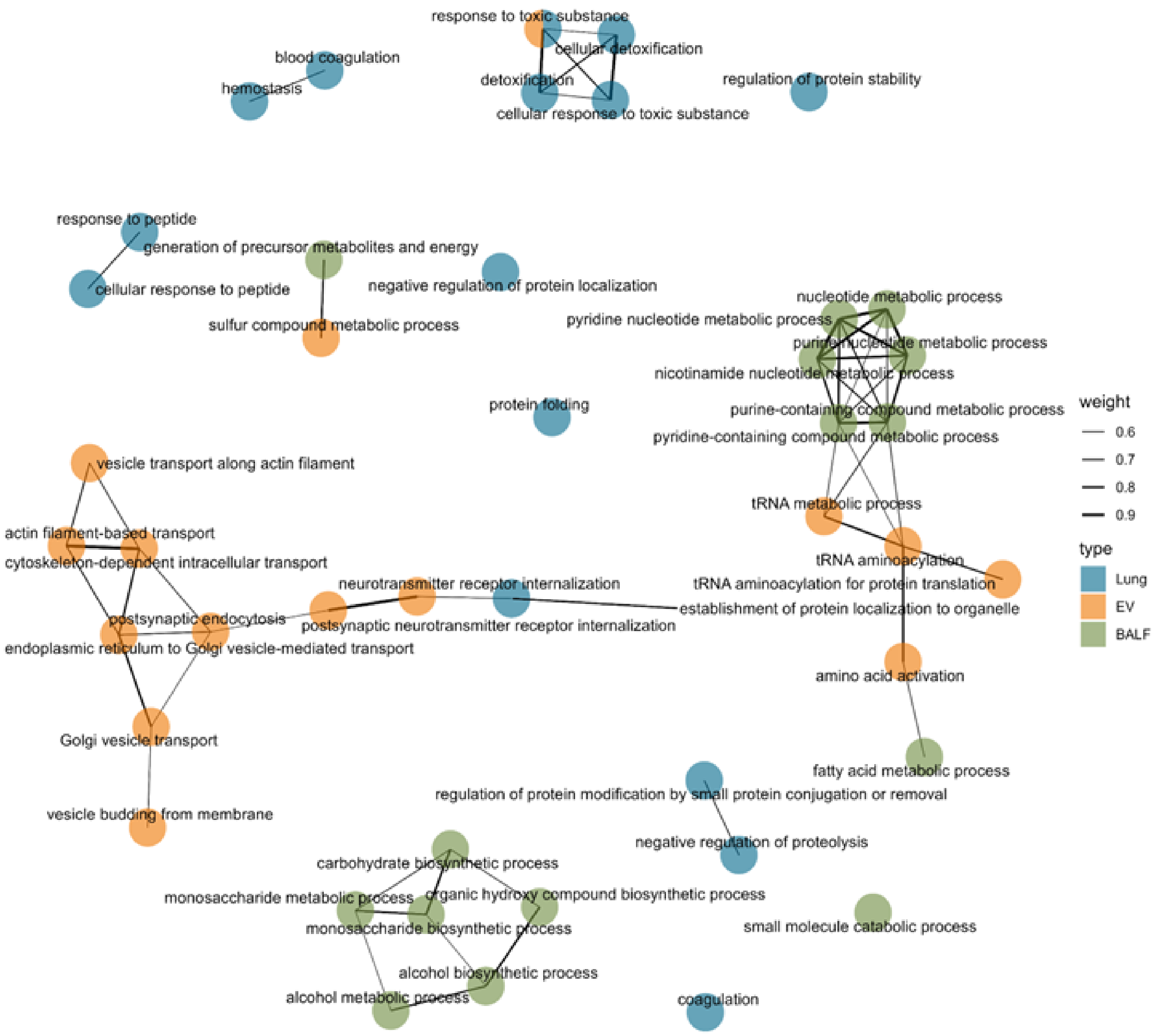

Supp. Fig 3. Labelled network analysis of top 15 downregulated pathways for each compartment, with nodes representing pathways and edges indicating shared functional relationships.

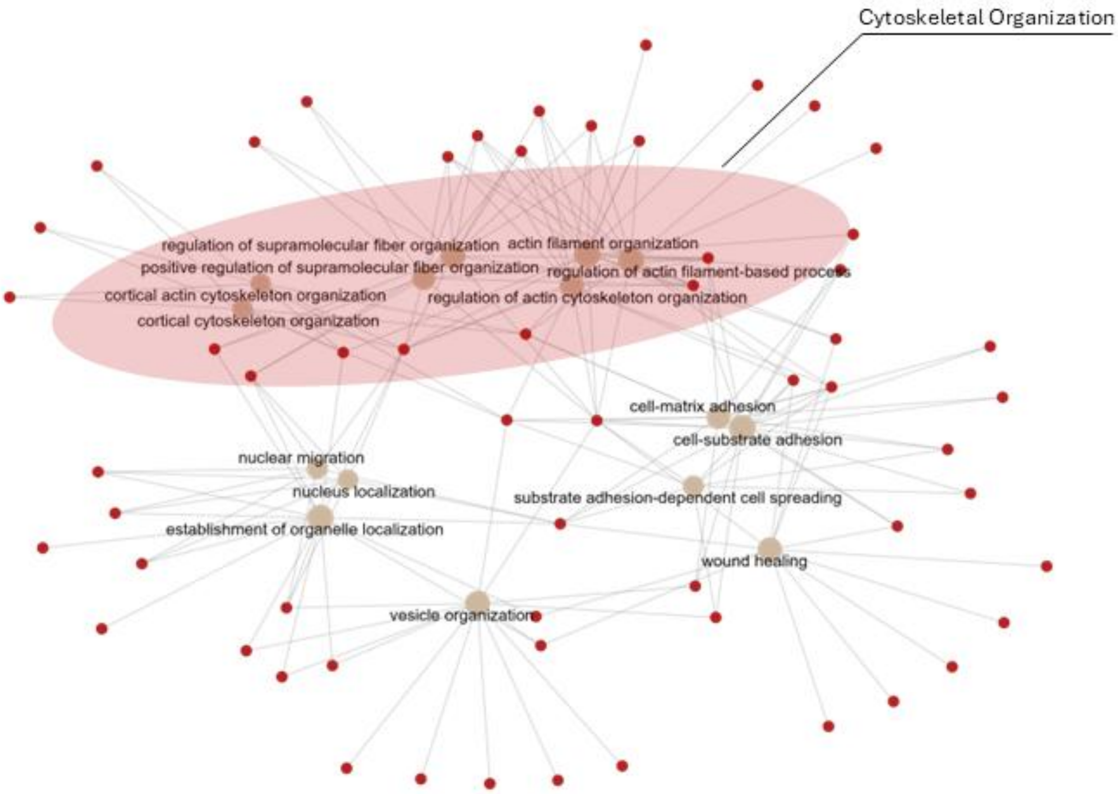

Supp. Fig 4. Labelled network analysis of top 15 upregulated pathways in lung tissue of *Ahr*$^{+/-}$ mice. Beige nodes represent enriched GO biological processes, while red nodes indicate associated proteins.

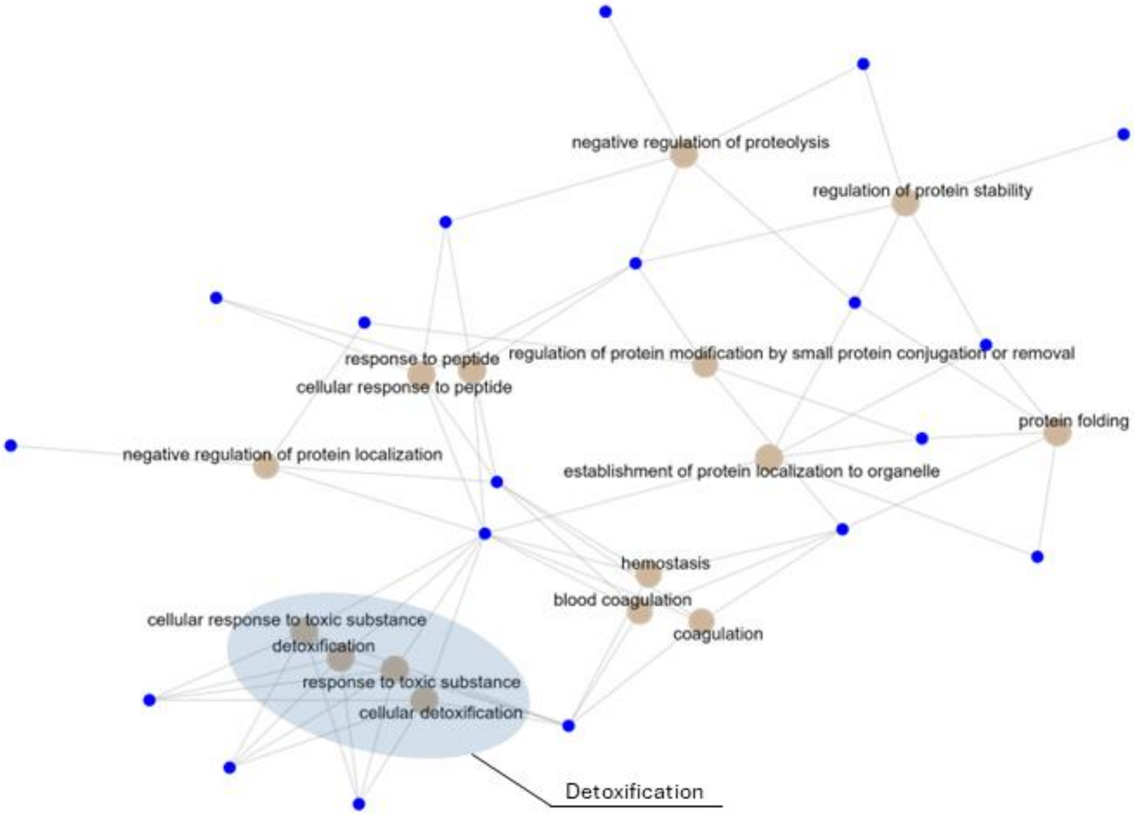

Supp. Fig 5. Labelled network analysis of top 15 downregulated pathways in lung tissue of *Ahr*$^{+/-}$ mice. Beige nodes represent enriched GO biological processes, while blue nodes indicate associated proteins.

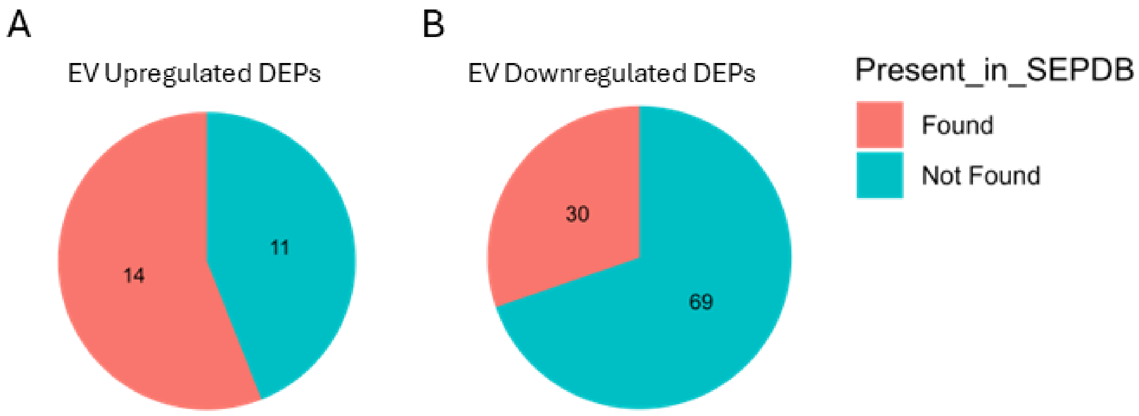

Supp. Fig 6. Proportion of EV DEPs upregulated (A) and downregulated (B) by AhR identified in the SEPDB.

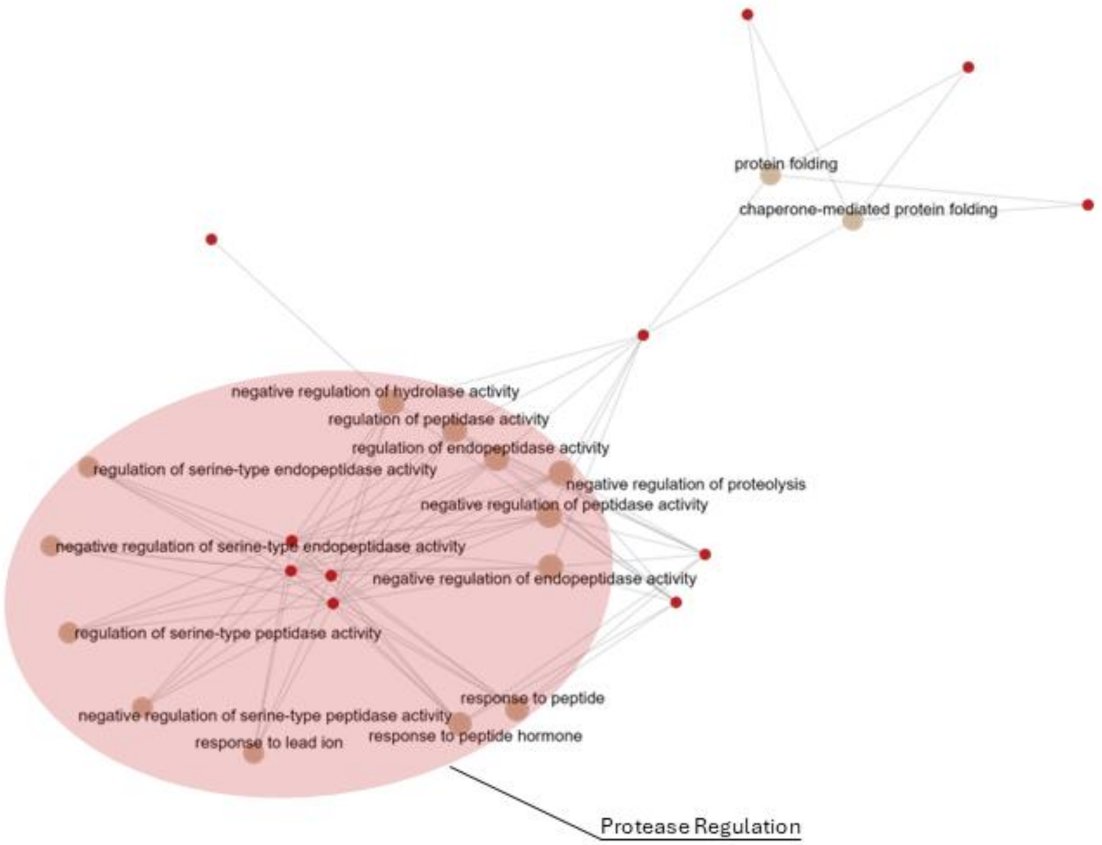

Supp. Fig 7. Labelled network analysis of top 15 upregulated pathways in EVs of *Ahr*$^{+/-}$ mice. Beige nodes represent enriched GO biological processes, while red nodes indicate associated proteins.

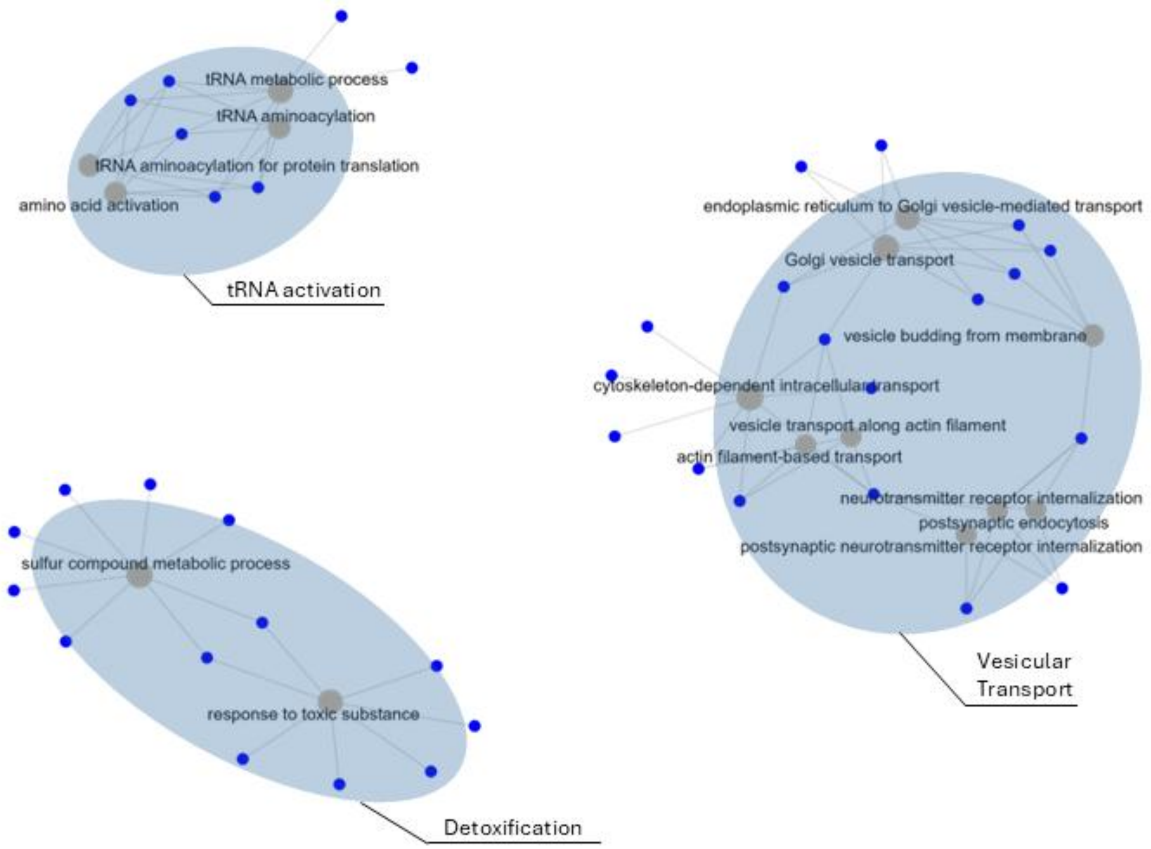

Supp. Fig 8. Labelled network analysis of top 15 downregulated pathways in EVs of *Ahr*$^{+/-}$ mice. Beige nodes represent enriched GO biological processes, while blue nodes indicate associated proteins.

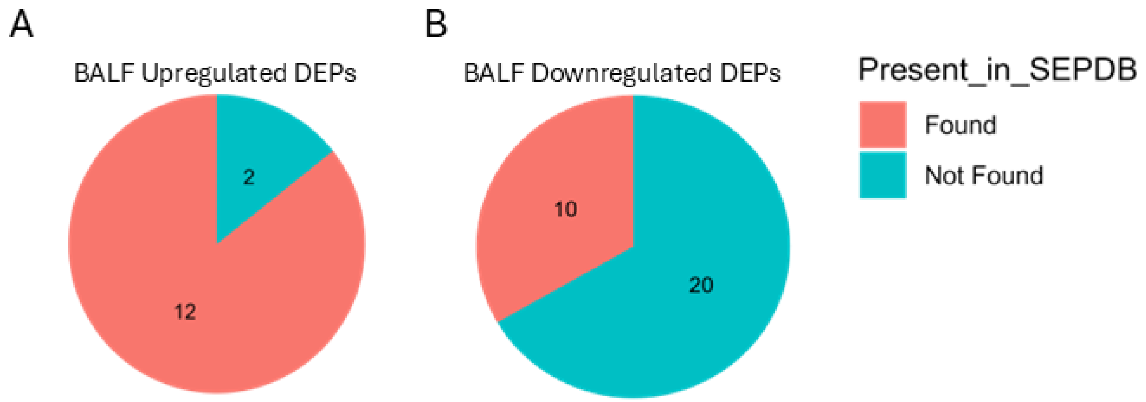

Supp. Fig 9. Proportion of BALF DEPs upregulated (A) and downregulated (B) by AhR identified in the SEPDB.

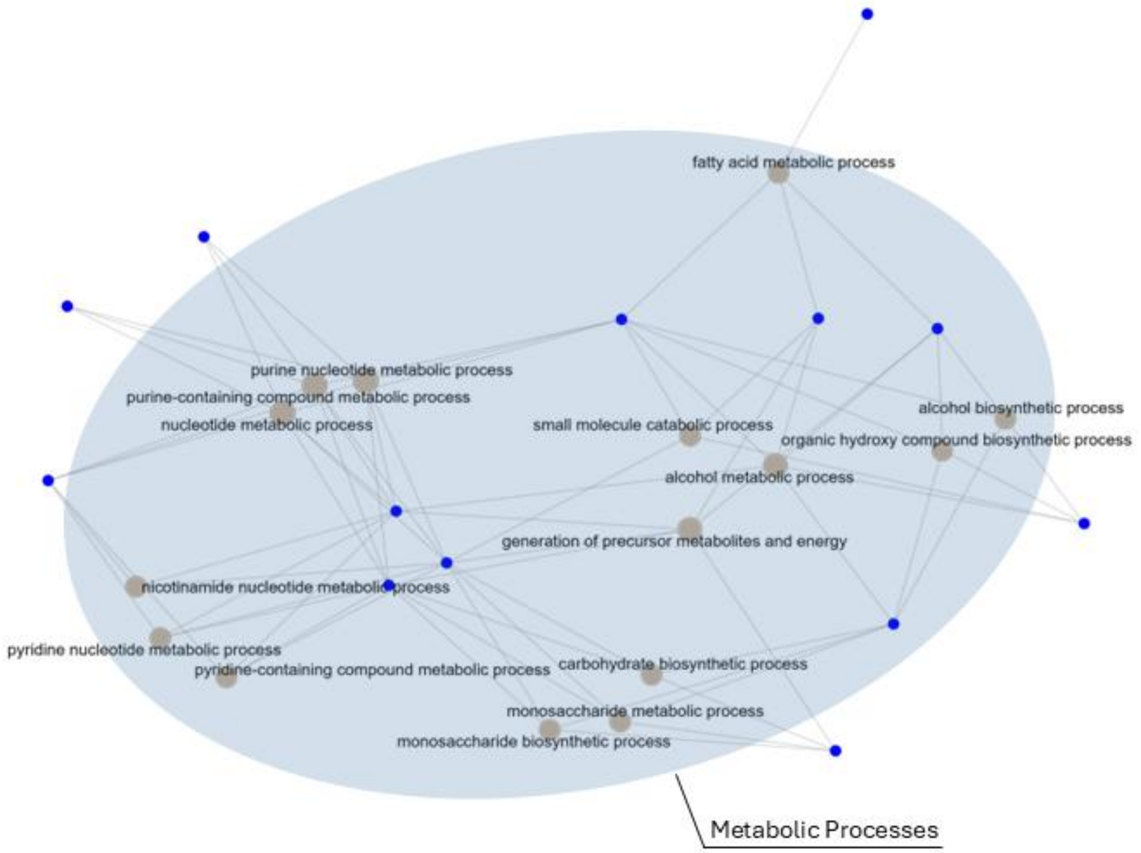

Supp. Fig 10. Labelled network analysis of top 15 downregulated pathways in BALF of *Ahr*$^{+/-}$ mice. Beige nodes represent enriched GO biological processes, while blue nodes indicate associated proteins.
